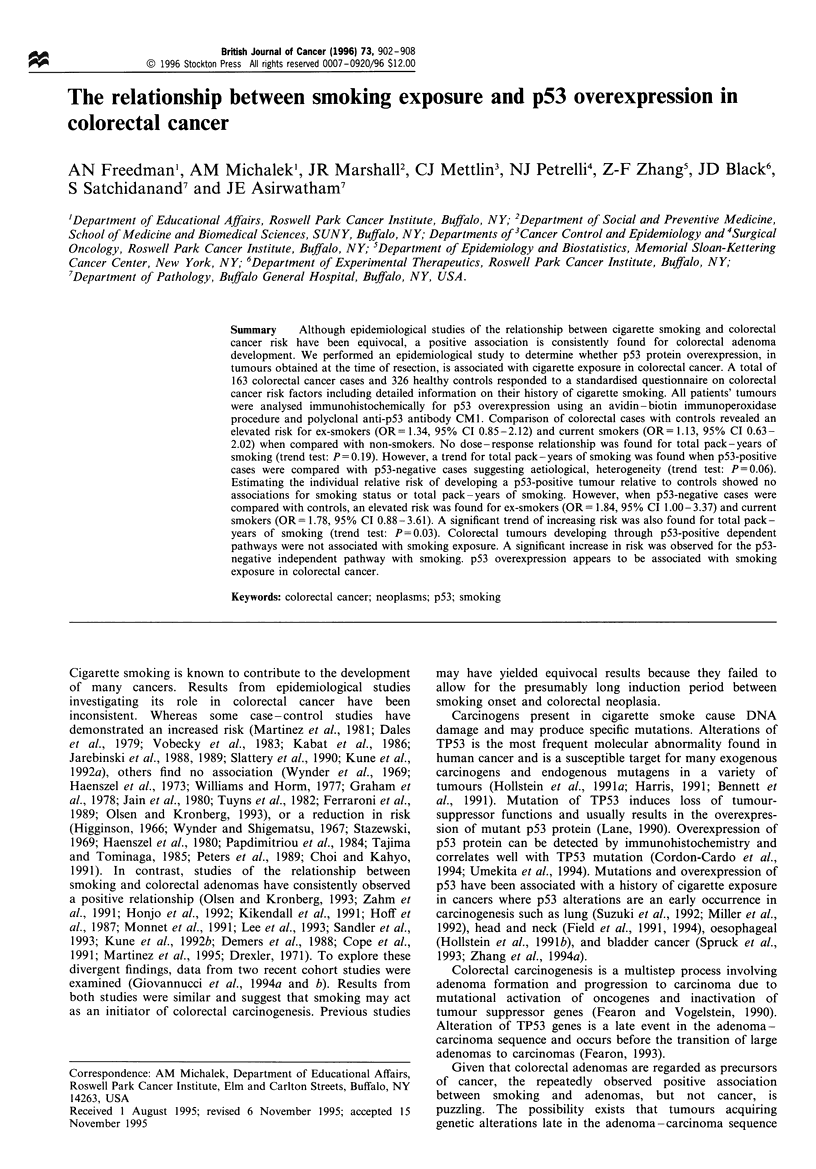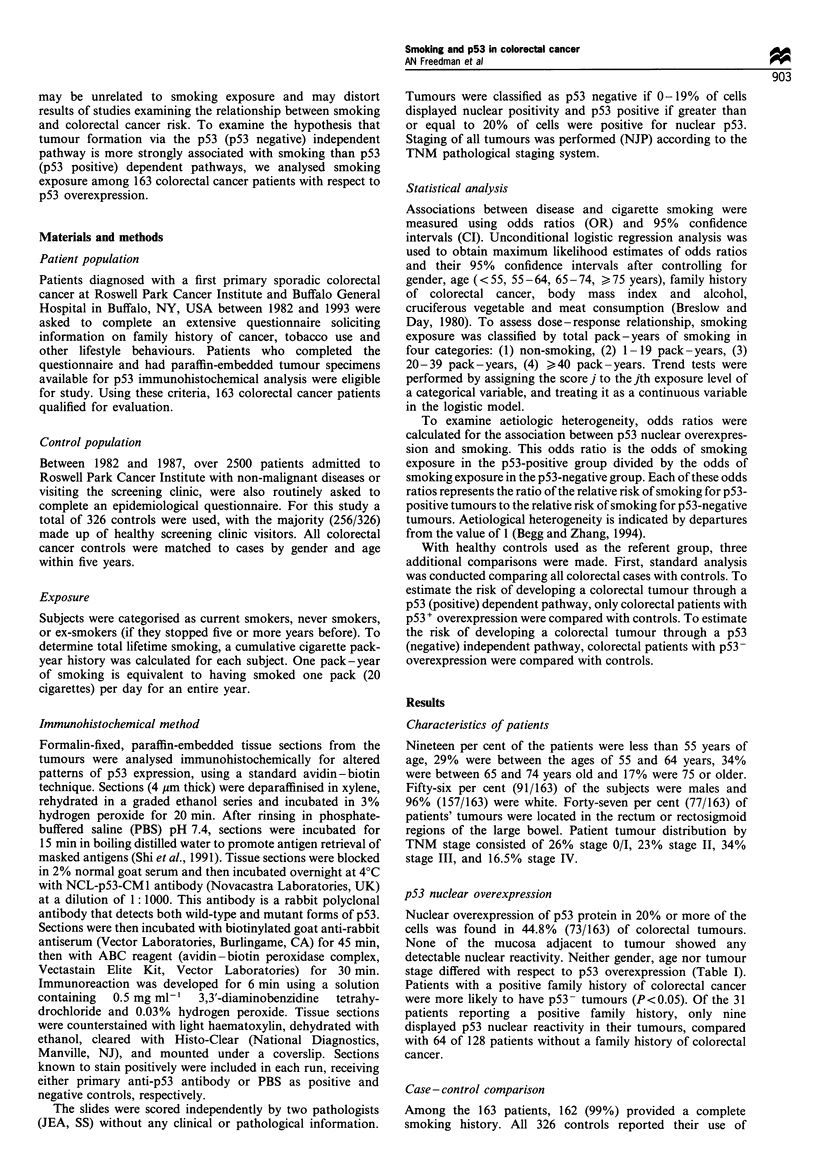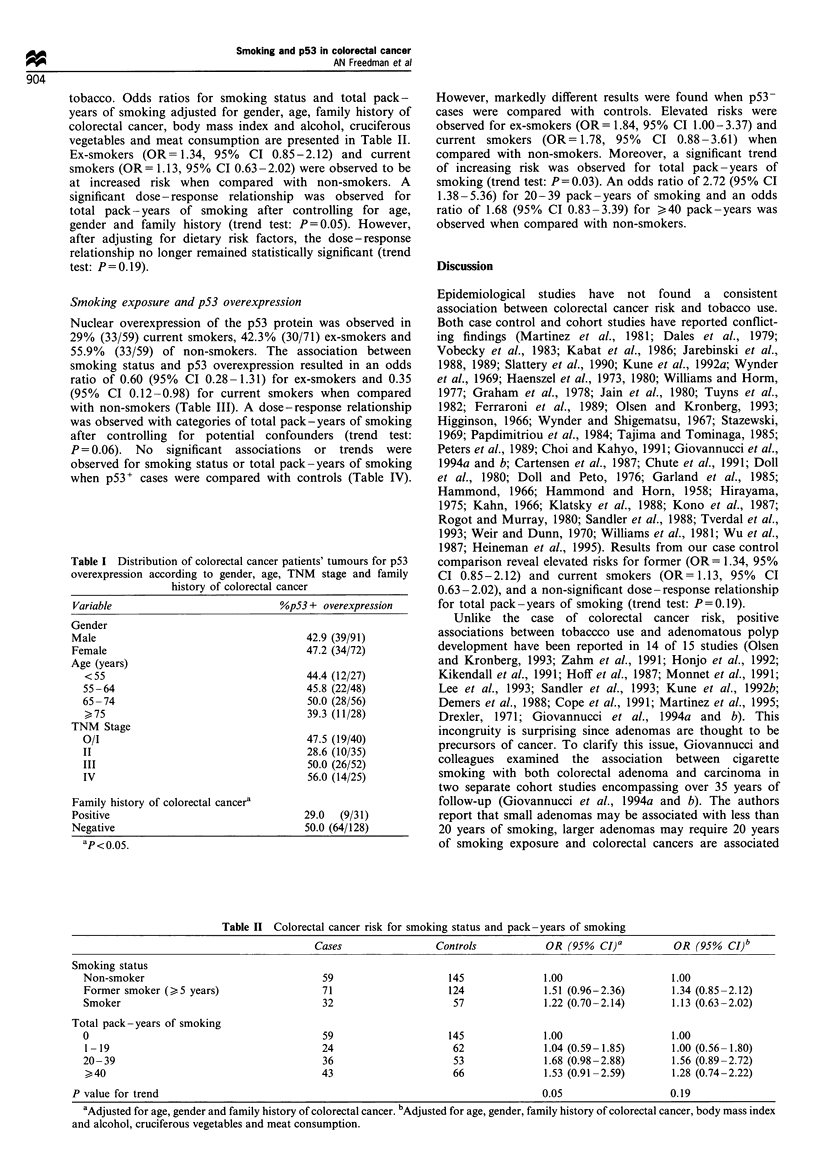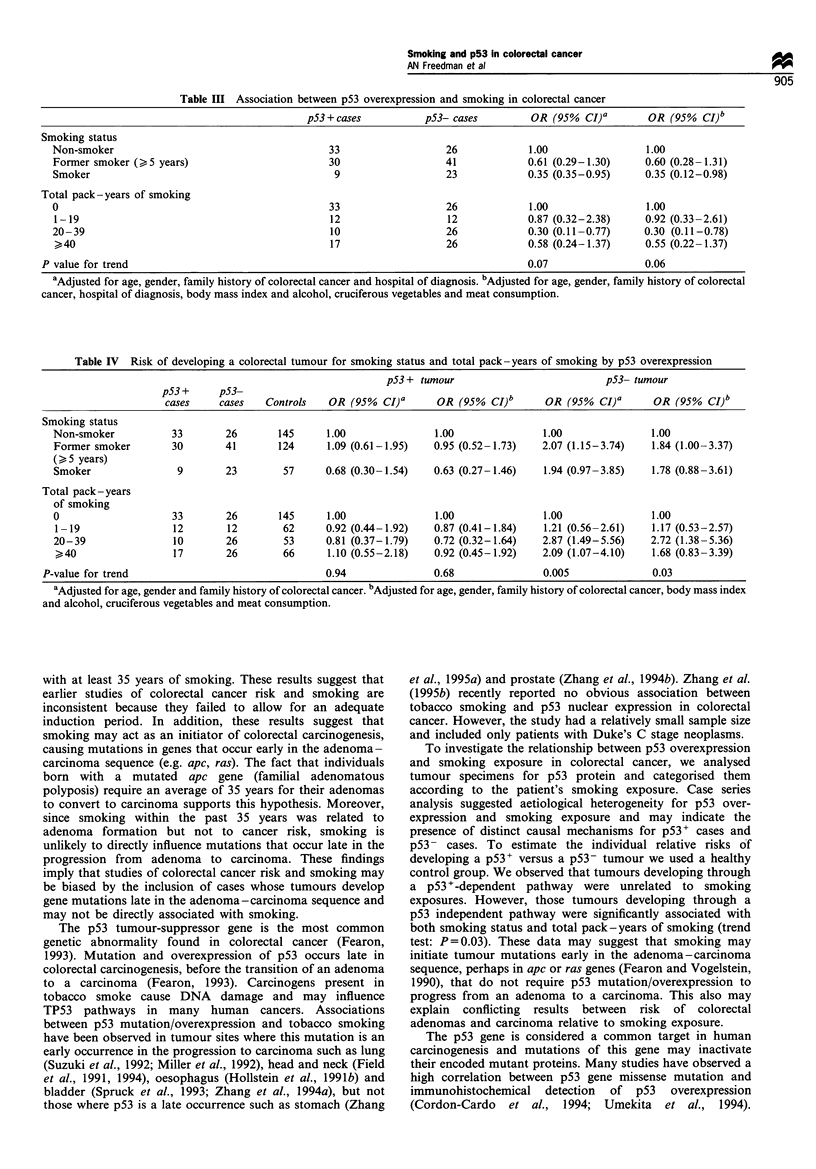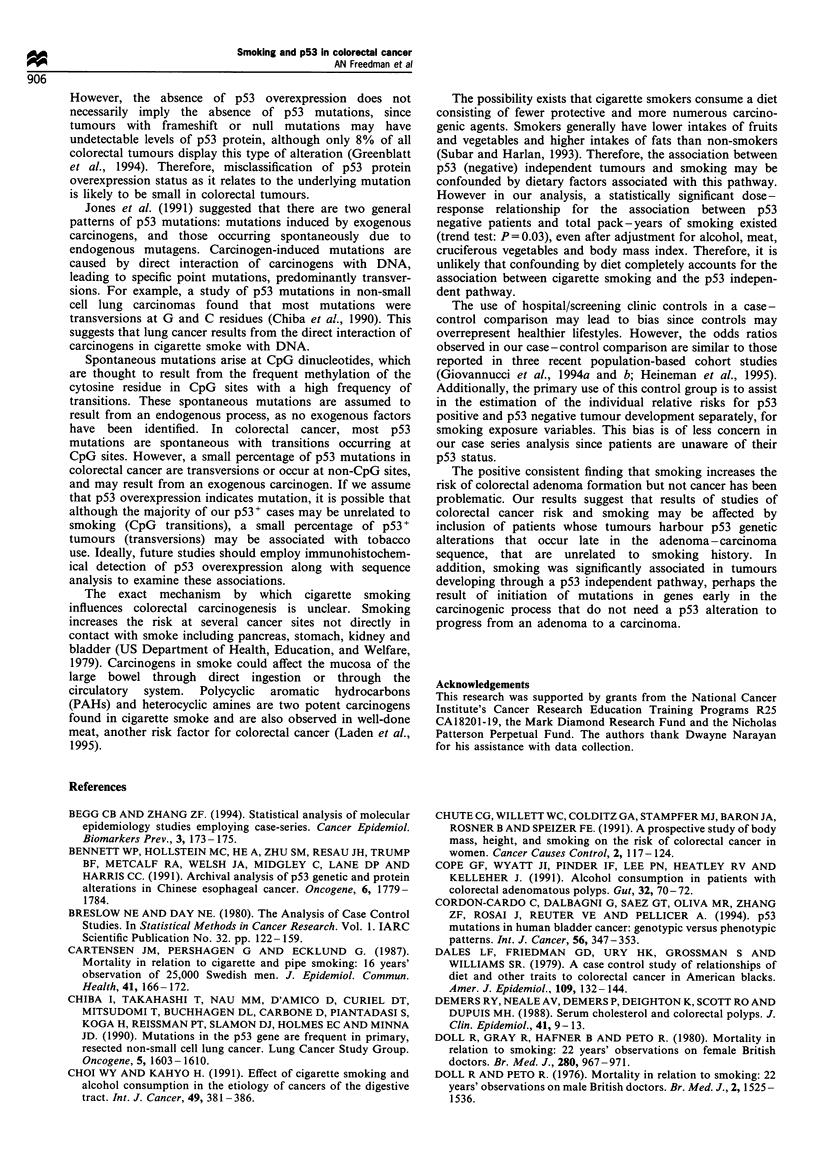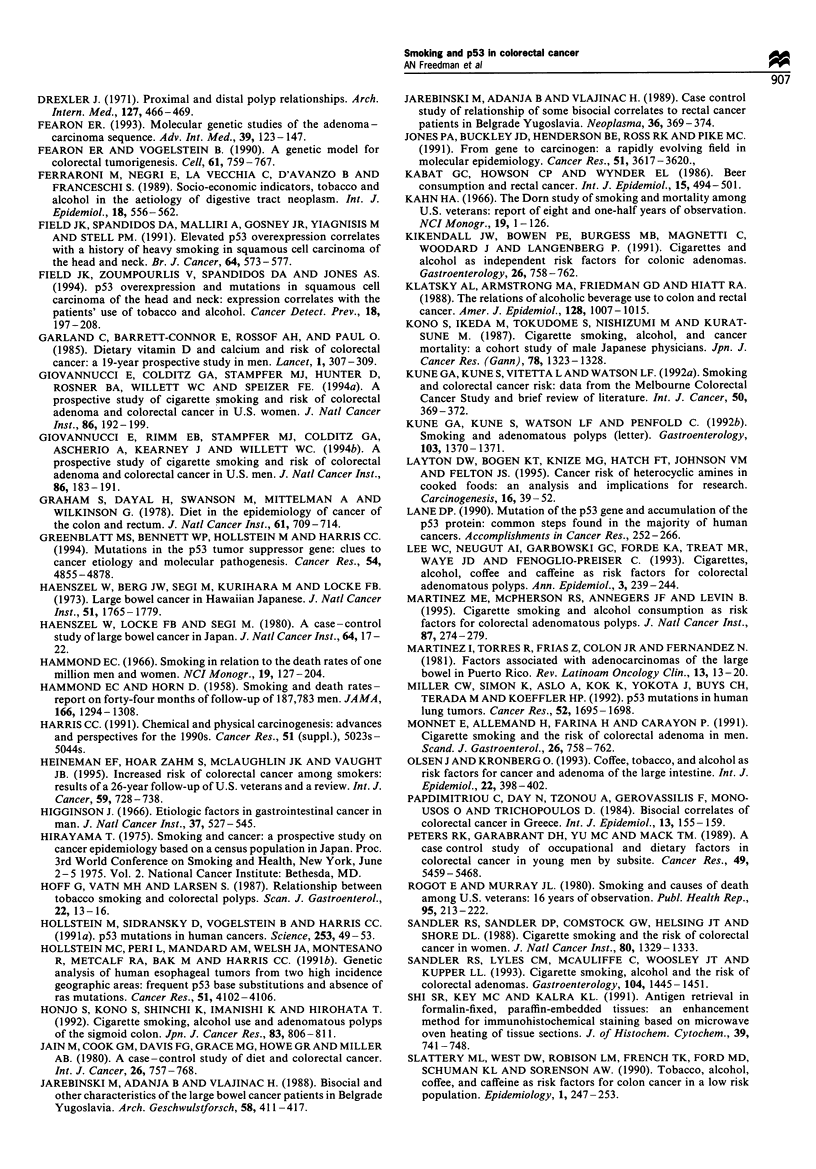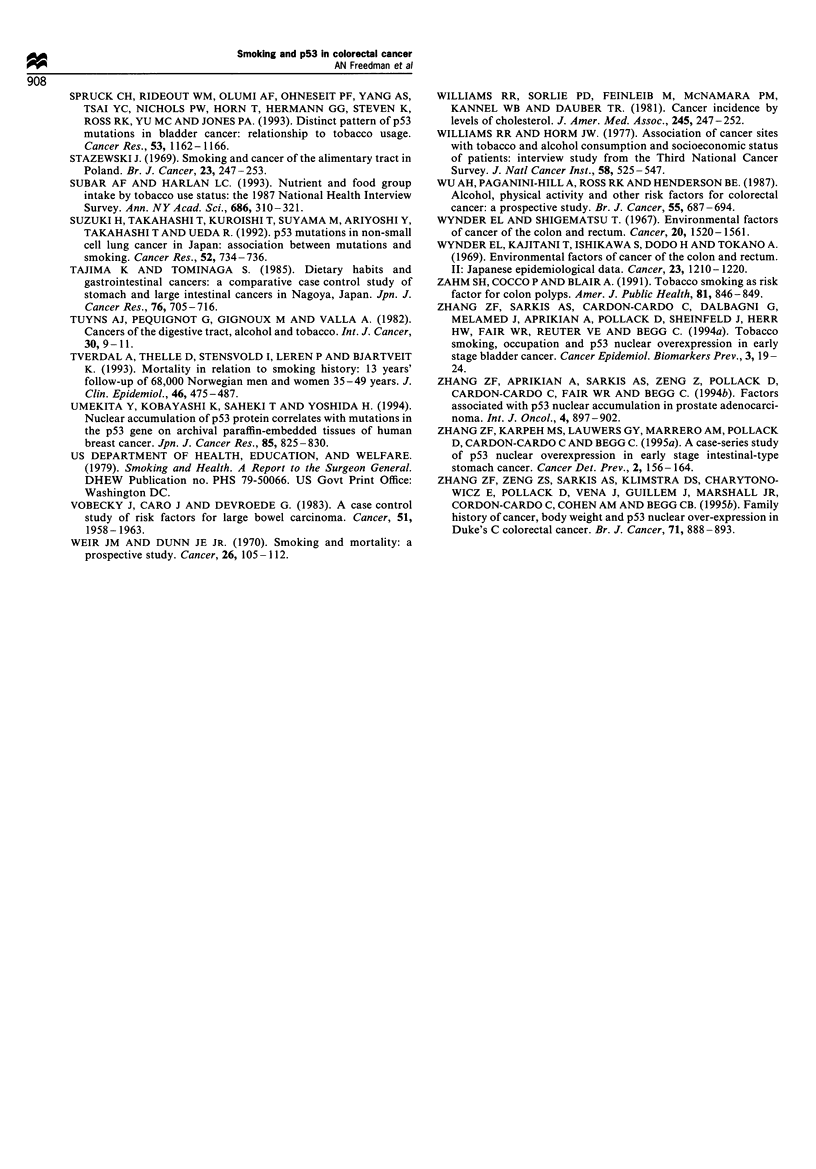# The relationship between smoking exposure and p53 overexpression in colorectal cancer.

**DOI:** 10.1038/bjc.1996.180

**Published:** 1996-04

**Authors:** A. N. Freedman, A. M. Michalek, J. R. Marshall, C. J. Mettlin, N. J. Petrelli, Z. F. Zhang, J. D. Black, S. Satchidanand, J. E. Asirwatham

**Affiliations:** Department of Educational Affairs, Roswell Park Cancer Institute, Buffalo, NY 14263, USA.

## Abstract

Although epidemiological studies of the relationship between cigarette smoking and colorectal cancer risk have been equivocal, a positive association is consistently found for colorectal adenoma development. We performed an epidemiological study to determine whether p53 protein overexpression, in tumours obtained at the time of resection, is associated with cigarette exposure in colorectal cancer. A total of 163 colorectal cancer cases and 326 healthy controls responded to a standardised questionnaire on colorectal cancer risk factors including detailed information on their history of cigarette smoking. All patients' tumours were analysed immunohistochemically for p53 overexpression using an avidin-biotin immunoperoxidase procedure and polyclonal anti-p53 antibody CM1. Comparison of colorectal cases with controls revealed an elevated risk for ex-smokers (OR = 1.34, 95% CI 0.85-2.12) and current smokers (OR = 1.13, 95% CI 0.63-2.02) when compared with non-smokers. No dose-response relationship was found for total pack-years of smoking (trend test: P = 0.19). However, a trend for total pack-years of smoking was found when p53-positive cases were compared with p53-negative cases suggesting aetiological, heterogeneity (trend test: P = 0.06). Estimating the individual relative risk of developing a p53-positive tumour relative to controls showed no associations for smoking status or total pack-years of smoking. However, when p53-negative cases were compared with controls, an elevated risk was found for ex-smokers (OR = 1.84, 95% CI 1.00-3.37) and current years of smoking (trend test: P = 0.03). Colorectal tumours developing through p53-positive dependent pathways were not associated with smoking exposure. A significant increase in risk was observed for the p53-negative independent pathway with smoking. p53 overexpression appears to be associated with smoking exposure in colorectal cancer.


British Journal of Cancer (1996) 73, 902-908
© 1996 Stockton Press All rights reserved 0007-0920/96 $12.00

### The relationship between smoking exposure and p53 overexpression in colorectal cancer

AN Freedman[1], AM Michalek[1], JR Marshall[2], CJ Mettlin[3], NJ Petrelli[4], Z-F Zhang[5], JD Black[6], S Satchidanand[7] and JE Asirwatham[7]

[1]*Department of Educational Affairs, Roswell Park Cancer Institute, Buffalo, NY;* [2]*Department of Social and Preventive Medicine, School of Medicine and Biomedical Sciences, SUNY, Buffalo, NY; Departments of* [3]*Cancer Control and Epidemiology and* [4]*Surgical Oncology, Roswell Park Cancer Institute, Buffalo, NY;* [5]*Department of Epidemiology and Biostatistics, Memorial Sloan-Kettering Cancer Center, New York, NY;* [6]*Department of Experimental Therapeutics, Roswell Park Cancer Institute, Buffalo, NY;* [7]*Department of Pathology, Buffalo General Hospital, Buffalo, NY, USA.*

**Summary** Although epidemiological studies of the relationship between cigarette smoking and colorectal cancer risk have been equivocal, a positive association is consistently found for colorectal adenoma development. We performed an epidemiological study to determine whether p53 protein overexpression, in tumours obtained at the time of resection, is associated with cigarette exposure in colorectal cancer. A total of 163 colorectal cancer cases and 326 healthy controls responded to a standardised questionnaire on colorectal cancer risk factors including detailed information on their history of cigarette smoking. All patients' tumours were analysed immunohistochemically for p53 overexpression using an avidin-biotin immunoperoxidase procedure and polyclonal anti-p53 antibody CM1. Comparison of colorectal cases with controls revealed an elevated risk for ex-smokers (OR = 1.34, 95% CI 0.85-2.12) and current smokers (OR = 1.13, 95% CI 0.63-2.02) when compared with non-smokers. No dose-response relationship was found for total pack-years of smoking (trend test: $P = 0.19$). However, a trend for total pack-years of smoking was found when p53-positive cases were compared with p53-negative cases suggesting aetiological, heterogeneity (trend test: $P = 0.06$). Estimating the individual relative risk of developing a p53-positive tumour relative to controls showed no associations for smoking status or total pack-years of smoking. However, when p53-negative cases were compared with controls, an elevated risk was found for ex-smokers (OR = 1.84, 95% CI 1.00-3.37) and current smokers (OR = 1.78, 95% CI 0.88-3.61). A significant trend of increasing risk was also found for total pack-years of smoking (trend test: $P = 0.03$). Colorectal tumours developing through p53-positive dependent pathways were not associated with smoking exposure. A significant increase in risk was observed for the p53-negative independent pathway with smoking. p53 overexpression appears to be associated with smoking exposure in colorectal cancer.

**Keywords:** colorectal cancer; neoplasms; p53; smoking

Cigarette smoking is known to contribute to the development of many cancers. Results from epidemiological studies investigating its role in colorectal cancer have been inconsistent. Whereas some case-control studies have demonstrated an increased risk (Martinez *et al.*, 1981; Dales *et al.*, 1979; Vobecky *et al.*, 1983; Kabat *et al.*, 1986; Jarebinski *et al.*, 1988, 1989; Slattery *et al.*, 1990; Kune *et al.*, 1992*a*), others find no association (Wynder *et al.*, 1969; Haenszel *et al.*, 1973; Williams and Horm, 1977; Graham *et al.*, 1978; Jain *et al.*, 1980; Tuyns *et al.*, 1982; Ferraroni *et al.*, 1989; Olsen and Kronberg, 1993), or a reduction in risk (Higginson, 1966; Wynder and Shigematsu, 1967; Stazewski, 1969; Haenszel *et al.*, 1980; Papdimitriou *et al.*, 1984; Tajima and Tominaga, 1985; Peters *et al.*, 1989; Choi and Kahyo, 1991). In contrast, studies of the relationship between smoking and colorectal adenomas have consistently observed a positive relationship (Olsen and Kronberg, 1993; Zahm *et al.*, 1991; Honjo *et al.*, 1992; Kikendall *et al.*, 1991; Hoff *et al.*, 1987; Monnet *et al.*, 1991; Lee *et al.*, 1993; Sandler *et al.*, 1993; Kune *et al.*, 1992*b*; Demers *et al.*, 1988; Cope *et al.*, 1991; Martinez *et al.*, 1995; Drexler, 1971). To explore these divergent findings, data from two recent cohort studies were examined (Giovannucci *et al.*, 1994*a* and *b*). Results from both studies were similar and suggest that smoking may act as an initiator of colorectal carcinogenesis. Previous studies may have yielded equivocal results because they failed to allow for the presumably long induction period between smoking onset and colorectal neoplasia.

Carcinogens present in cigarette smoke cause DNA damage and may produce specific mutations. Alterations of TP53 is the most frequent molecular abnormality found in human cancer and is a susceptible target for many exogenous carcinogens and endogenous mutagens in a variety of tumours (Hollstein *et al.*, 1991*a*; Harris, 1991; Bennett *et al.*, 1991). Mutation of TP53 induces loss of tumour-suppressor functions and usually results in the overexpression of mutant p53 protein (Lane, 1990). Overexpression of p53 protein can be detected by immunohistochemistry and correlates well with TP53 mutation (Cordon-Cardo *et al.*, 1994; Umekita *et al.*, 1994). Mutations and overexpression of p53 have been associated with a history of cigarette exposure in cancers where p53 alterations are an early occurrence in carcinogenesis such as lung (Suzuki *et al.*, 1992; Miller *et al.*, 1992), head and neck (Field *et al.*, 1991, 1994), oesophageal (Hollstein *et al.*, 1991*b*), and bladder cancer (Spruck *et al.*, 1993; Zhang *et al.*, 1994*a*).

Colorectal carcinogenesis is a multistep process involving adenoma formation and progression to carcinoma due to mutational activation of oncogenes and inactivation of tumour suppressor genes (Fearon and Vogelstein, 1990). Alteration of TP53 genes is a late event in the adenoma-carcinoma sequence and occurs before the transition of large adenomas to carcinomas (Fearon, 1993).

Given that colorectal adenomas are regarded as precursors of cancer, the repeatedly observed positive association between smoking and adenomas, but not cancer, is puzzling. The possibility exists that tumours acquiring genetic alterations late in the adenoma-carcinoma sequence

Correspondence: AM Michalek, Department of Educational Affairs, Roswell Park Cancer Institute, Elm and Carlton Streets, Buffalo, NY 14263, USA
Received 1 August 1995; revised 6 November 1995; accepted 15 November 1995

may be unrelated to smoking exposure and may distort results of studies examining the relationship between smoking and colorectal cancer risk. To examine the hypothesis that tumour formation via the p53 (p53 negative) independent pathway is more strongly associated with smoking than p53 (p53 positive) dependent pathways, we analysed smoking exposure among 163 colorectal cancer patients with respect to p53 overexpression.

### Materials and methods

### *Patient population*

Patients diagnosed with a first primary sporadic colorectal cancer at Roswell Park Cancer Institute and Buffalo General Hospital in Buffalo, NY, USA between 1982 and 1993 were asked to complete an extensive questionnaire soliciting information on family history of cancer, tobacco use and other lifestyle behaviours. Patients who completed the questionnaire and had paraffin-embedded tumour specimens available for p53 immunohistochemical analysis were eligible for study. Using these criteria, 163 colorectal cancer patients qualified for evaluation.

### *Control population*

Between 1982 and 1987, over 2500 patients admitted to Roswell Park Cancer Institute with non-malignant diseases or visiting the screening clinic, were also routinely asked to complete an epidemiological questionnaire. For this study a total of 326 controls were used, with the majority (256/326) made up of healthy screening clinic visitors. All colorectal cancer controls were matched to cases by gender and age within five years.

### *Exposure*

Subjects were categorised as current smokers, never smokers, or ex-smokers (if they stopped five or more years before). To determine total lifetime smoking, a cumulative cigarette pack-year history was calculated for each subject. One pack–year of smoking is equivalent to having smoked one pack (20 cigarettes) per day for an entire year.

### *Immunohistochemical method*

Formalin-fixed, paraffin-embedded tissue sections from the tumours were analysed immunohistochemically for altered patterns of p53 expression, using a standard avidin–biotin technique. Sections (4 $\mu$m thick) were deparaffinised in xylene, rehydrated in a graded ethanol series and incubated in 3% hydrogen peroxide for 20 min. After rinsing in phosphate-buffered saline (PBS) pH 7.4, sections were incubated for 15 min in boiling distilled water to promote antigen retrieval of masked antigens (Shi *et al*., 1991). Tissue sections were blocked in 2% normal goat serum and then incubated overnight at 4°C with NCL-p53-CM1 antibody (Novacastra Laboratories, UK) at a dilution of 1:1000. This antibody is a rabbit polyclonal antibody that detects both wild-type and mutant forms of p53. Sections were then incubated with biotinylated goat anti-rabbit antiserum (Vector Laboratories, Burlingame, CA) for 45 min, then with ABC reagent (avidin–biotin peroxidase complex, Vectastain Elite Kit, Vector Laboratories) for 30 min. Immunoreaction was developed for 6 min using a solution containing 0.5 mg $ml^{-1}$ 3,3′-diaminobenzidine tetrahydrochloride and 0.03% hydrogen peroxide. Tissue sections were counterstained with light haematoxylin, dehydrated with ethanol, cleared with Histo-Clear (National Diagnostics, Manville, NJ), and mounted under a coverslip. Sections known to stain positively were included in each run, receiving either primary anti-p53 antibody or PBS as positive and negative controls, respectively.

The slides were scored independently by two pathologists (JEA, SS) without any clinical or pathological information. Tumours were classified as p53 negative if 0–19% of cells displayed nuclear positivity and p53 positive if greater than or equal to 20% of cells were positive for nuclear p53. Staging of all tumours was performed (NJP) according to the TNM pathological staging system.

### *Statistical analysis*

Associations between disease and cigarette smoking were measured using odds ratios (OR) and 95% confidence intervals (CI). Unconditional logistic regression analysis was used to obtain maximum likelihood estimates of odds ratios and their 95% confidence intervals after controlling for gender, age (<55, 55–64, 65–74, ⩾75 years), family history of colorectal cancer, body mass index and alcohol, cruciferous vegetable and meat consumption (Breslow and Day, 1980). To assess dose–response relationship, smoking exposure was classified by total pack–years of smoking in four categories: (1) non-smoking, (2) 1–19 pack–years, (3) 20–39 pack–years, (4) ⩾40 pack–years. Trend tests were performed by assigning the score $j$ to the $j$th exposure level of a categorical variable, and treating it as a continuous variable in the logistic model.

To examine aetiologic heterogeneity, odds ratios were calculated for the association between p53 nuclear overexpression and smoking. This odds ratio is the odds of smoking exposure in the p53-positive group divided by the odds of smoking exposure in the p53-negative group. Each of these odds ratios represents the ratio of the relative risk of smoking for p53-positive tumours to the relative risk of smoking for p53-negative tumours. Aetiological heterogeneity is indicated by departures from the value of 1 (Begg and Zhang, 1994).

With healthy controls used as the referent group, three additional comparisons were made. First, standard analysis was conducted comparing all colorectal cases with controls. To estimate the risk of developing a colorectal tumour through a p53 (positive) dependent pathway, only colorectal patients with $p53^{+}$ overexpression were compared with controls. To estimate the risk of developing a colorectal tumour through a p53 (negative) independent pathway, colorectal patients with $p53^{-}$ overexpression were compared with controls.

### Results

### *Characteristics of patients*

Nineteen per cent of the patients were less than 55 years of age, 29% were between the ages of 55 and 64 years, 34% were between 65 and 74 years old and 17% were 75 or older. Fifty-six per cent (91/163) of the subjects were males and 96% (157/163) were white. Forty-seven per cent (77/163) of patients' tumours were located in the rectum or rectosigmoid regions of the large bowel. Patient tumour distribution by TNM stage consisted of 26% stage 0/I, 23% stage II, 34% stage III, and 16.5% stage IV.

### *p53 nuclear overexpression*

Nuclear overexpression of p53 protein in 20% or more of the cells was found in 44.8% (73/163) of colorectal tumours. None of the mucosa adjacent to tumour showed any detectable nuclear reactivity. Neither gender, age nor tumour stage differed with respect to p53 overexpression (Table I). Patients with a positive family history of colorectal cancer were more likely to have $p53^{-}$ tumours ($P<0.05$). Of the 31 patients reporting a positive family history, only nine displayed p53 nuclear reactivity in their tumours, compared with 64 of 128 patients without a family history of colorectal cancer.

### *Case–control comparison*

Among the 163 patients, 162 (99%) provided a complete smoking history. All 326 controls reported their use of tobacco. Odds ratios for smoking status and total pack-years of smoking adjusted for gender, age, family history of colorectal cancer, body mass index and alcohol, cruciferous vegetables and meat consumption are presented in Table II. Ex-smokers (OR = 1.34, 95% CI 0.85-2.12) and current smokers (OR = 1.13, 95% CI 0.63-2.02) were observed to be at increased risk when compared with non-smokers. A significant dose-response relationship was observed for total pack-years of smoking after controlling for age, gender and family history (trend test: $P = 0.05$). However, after adjusting for dietary risk factors, the dose-response relationship no longer remained statistically significant (trend test: $P = 0.19$).

*Smoking exposure and p53 overexpression*

Nuclear overexpression of the p53 protein was observed in 29% (33/59) current smokers, 42.3% (30/71) ex-smokers and 55.9% (33/59) of non-smokers. The association between smoking status and p53 overexpression resulted in an odds ratio of 0.60 (95% CI 0.28-1.31) for ex-smokers and 0.35 (95% CI 0.12-0.98) for current smokers when compared with non-smokers (Table III). A dose-response relationship was observed with categories of total pack-years of smoking after controlling for potential confounders (trend test: $P = 0.06$). No significant associations or trends were observed for smoking status or total pack-years of smoking when $p53^+$ cases were compared with controls (Table IV).

However, markedly different results were found when $p53^-$ cases were compared with controls. Elevated risks were observed for ex-smokers (OR = 1.84, 95% CI 1.00-3.37) and current smokers (OR = 1.78, 95% CI 0.88-3.61) when compared with non-smokers. Moreover, a significant trend of increasing risk was observed for total pack-years of smoking (trend test: $P = 0.03$). An odds ratio of 2.72 (95% CI 1.38-5.36) for 20-39 pack-years of smoking and an odds ratio of 1.68 (95% CI 0.83-3.39) for ⩾40 pack-years was observed when compared with non-smokers.

**Table I** Distribution of colorectal cancer patients' tumours for p53 overexpression according to gender, age, TNM stage and family history of colorectal cancer

| *Variable* | *%p53+ overexpression* |
|---|---|
| Gender | |
| Male | 42.9 (39/91) |
| Female | 47.2 (34/72) |
| Age (years) | |
| <55 | 44.4 (12/27) |
| 55-64 | 45.8 (22/48) |
| 65-74 | 50.0 (28/56) |
| ⩾75 | 39.3 (11/28) |
| TNM Stage | |
| O/I | 47.5 (19/40) |
| II | 28.6 (10/35) |
| III | 50.0 (26/52) |
| IV | 56.0 (14/25) |
| Family history of colorectal cancer[a] | |
| Positive | 29.0 (9/31) |
| Negative | 50.0 (64/128) |

[a] $P < 0.05$.

### Discussion

Epidemiological studies have not found a consistent association between colorectal cancer risk and tobacco use. Both case control and cohort studies have reported conflicting findings (Martinez *et al.*, 1981; Dales *et al.*, 1979; Vobecky *et al.*, 1983; Kabat *et al.*, 1986; Jarebinski *et al.*, 1988, 1989; Slattery *et al.*, 1990; Kune *et al.*, 1992*a*; Wynder *et al.*, 1969; Haenszel *et al.*, 1973, 1980; Williams and Horm, 1977; Graham *et al.*, 1978; Jain *et al.*, 1980; Tuyns *et al.*, 1982; Ferraroni *et al.*, 1989; Olsen and Kronberg, 1993; Higginson, 1966; Wynder and Shigematsu, 1967; Stazewski, 1969; Papdimitriou *et al.*, 1984; Tajima and Tominaga, 1985; Peters *et al.*, 1989; Choi and Kahyo, 1991; Giovannucci *et al.*, 1994*a* and *b*; Cartensen *et al.*, 1987; Chute *et al.*, 1991; Doll *et al.*, 1980; Doll and Peto, 1976; Garland *et al.*, 1985; Hammond, 1966; Hammond and Horn, 1958; Hirayama, 1975; Kahn, 1966; Klatsky *et al.*, 1988; Kono *et al.*, 1987; Rogot and Murray, 1980; Sandler *et al.*, 1988; Tverdal *et al.*, 1993; Weir and Dunn, 1970; Williams *et al.*, 1981; Wu *et al.*, 1987; Heineman *et al.*, 1995). Results from our case control comparison reveal elevated risks for former (OR = 1.34, 95% CI 0.85-2.12) and current smokers (OR = 1.13, 95% CI 0.63-2.02), and a non-significant dose-response relationship for total pack-years of smoking (trend test: $P = 0.19$).

Unlike the case of colorectal cancer risk, positive associations between tobaccco use and adenomatous polyp development have been reported in 14 of 15 studies (Olsen and Kronberg, 1993; Zahm *et al.*, 1991; Honjo *et al.*, 1992; Kikendall *et al.*, 1991; Hoff *et al.*, 1987; Monnet *et al.*, 1991; Lee *et al.*, 1993; Sandler *et al.*, 1993; Kune *et al.*, 1992*b*; Demers *et al.*, 1988; Cope *et al.*, 1991; Martinez *et al.*, 1995; Drexler, 1971; Giovannucci *et al.*, 1994*a* and *b*). This incongruity is surprising since adenomas are thought to be precursors of cancer. To clarify this issue, Giovannucci and colleagues examined the association between cigarette smoking with both colorectal adenoma and carcinoma in two separate cohort studies encompassing over 35 years of follow-up (Giovannucci *et al.*, 1994*a* and *b*). The authors report that small adenomas may be associated with less than 20 years of smoking, larger adenomas may require 20 years of smoking exposure and colorectal cancers are associated

**Table II** Colorectal cancer risk for smoking status and pack-years of smoking

| | *Cases* | *Controls* | *OR (95% CI)[a]* | *OR (95% CI)[b]* |
|---|---|---|---|---|
| Smoking status | | | | |
| Non-smoker | 59 | 145 | 1.00 | 1.00 |
| Former smoker (⩾5 years) | 71 | 124 | 1.51 (0.96-2.36) | 1.34 (0.85-2.12) |
| Smoker | 32 | 57 | 1.22 (0.70-2.14) | 1.13 (0.63-2.02) |
| Total pack-years of smoking | | | | |
| 0 | 59 | 145 | 1.00 | 1.00 |
| 1-19 | 24 | 62 | 1.04 (0.59-1.85) | 1.00 (0.56-1.80) |
| 20-39 | 36 | 53 | 1.68 (0.98-2.88) | 1.56 (0.89-2.72) |
| ⩾40 | 43 | 66 | 1.53 (0.91-2.59) | 1.28 (0.74-2.22) |
| *P* value for trend | | | 0.05 | 0.19 |

[a]Adjusted for age, gender and family history of colorectal cancer. [b]Adjusted for age, gender, family history of colorectal cancer, body mass index and alcohol, cruciferous vegetables and meat consumption.

**Table III** Association between p53 overexpression and smoking in colorectal cancer

| | *p53+cases* | *p53– cases* | *OR (95% CI)[a]* | *OR (95% CI)[b]* |
|---|---|---|---|---|
| Smoking status | | | | |
| Non-smoker | 33 | 26 | 1.00 | 1.00 |
| Former smoker (≥5 years) | 30 | 41 | 0.61 (0.29–1.30) | 0.60 (0.28–1.31) |
| Smoker | 9 | 23 | 0.35 (0.35–0.95) | 0.35 (0.12–0.98) |
| Total pack–years of smoking | | | | |
| 0 | 33 | 26 | 1.00 | 1.00 |
| 1–19 | 12 | 12 | 0.87 (0.32–2.38) | 0.92 (0.33–2.61) |
| 20–39 | 10 | 26 | 0.30 (0.11–0.77) | 0.30 (0.11–0.78) |
| ≥40 | 17 | 26 | 0.58 (0.24–1.37) | 0.55 (0.22–1.37) |
| *P* value for trend | | | 0.07 | 0.06 |

[a]Adjusted for age, gender, family history of colorectal cancer and hospital of diagnosis. [b]Adjusted for age, gender, family history of colorectal cancer, hospital of diagnosis, body mass index and alcohol, cruciferous vegetables and meat consumption.

**Table IV** Risk of developing a colorectal tumour for smoking status and total pack–years of smoking by p53 overexpression

| | | | | *p53+ tumour* | | *p53– tumour* | |
|---|---|---|---|---|---|---|---|
| | *p53+ cases* | *p53– cases* | *Controls* | *OR (95% CI)[a]* | *OR (95% CI)[b]* | *OR (95% CI)[a]* | *OR (95% CI)[b]* |
| Smoking status | | | | | | | |
| Non-smoker | 33 | 26 | 145 | 1.00 | 1.00 | 1.00 | 1.00 |
| Former smoker (≥5 years) | 30 | 41 | 124 | 1.09 (0.61–1.95) | 0.95 (0.52–1.73) | 2.07 (1.15–3.74) | 1.84 (1.00–3.37) |
| Smoker | 9 | 23 | 57 | 0.68 (0.30–1.54) | 0.63 (0.27–1.46) | 1.94 (0.97–3.85) | 1.78 (0.88–3.61) |
| Total pack–years of smoking | | | | | | | |
| 0 | 33 | 26 | 145 | 1.00 | 1.00 | 1.00 | 1.00 |
| 1–19 | 12 | 12 | 62 | 0.92 (0.44–1.92) | 0.87 (0.41–1.84) | 1.21 (0.56–2.61) | 1.17 (0.53–2.57) |
| 20–39 | 10 | 26 | 53 | 0.81 (0.37–1.79) | 0.72 (0.32–1.64) | 2.87 (1.49–5.56) | 2.72 (1.38–5.36) |
| ≥40 | 17 | 26 | 66 | 1.10 (0.55–2.18) | 0.92 (0.45–1.92) | 2.09 (1.07–4.10) | 1.68 (0.83–3.39) |
| *P*-value for trend | | | | 0.94 | 0.68 | 0.005 | 0.03 |

[a]Adjusted for age, gender and family history of colorectal cancer. [b]Adjusted for age, gender, family history of colorectal cancer, body mass index and alcohol, cruciferous vegetables and meat consumption.

with at least 35 years of smoking. These results suggest that earlier studies of colorectal cancer risk and smoking are inconsistent because they failed to allow for an adequate induction period. In addition, these results suggest that smoking may act as an initiator of colorectal carcinogenesis, causing mutations in genes that occur early in the adenoma–carcinoma sequence (e.g. *apc*, *ras*). The fact that individuals born with a mutated *apc* gene (familial adenomatous polyposis) require an average of 35 years for their adenomas to convert to carcinoma supports this hypothesis. Moreover, since smoking within the past 35 years was related to adenoma formation but not to cancer risk, smoking is unlikely to directly influence mutations that occur late in the progression from adenoma to carcinoma. These findings imply that studies of colorectal cancer risk and smoking may be biased by the inclusion of cases whose tumours develop gene mutations late in the adenoma–carcinoma sequence and may not be directly associated with smoking.

The p53 tumour-suppressor gene is the most common genetic abnormality found in colorectal cancer (Fearon, 1993). Mutation and overexpression of p53 occurs late in colorectal carcinogenesis, before the transition of an adenoma to a carcinoma (Fearon, 1993). Carcinogens present in tobacco smoke cause DNA damage and may influence TP53 pathways in many human cancers. Associations between p53 mutation/overexpression and tobacco smoking have been observed in tumour sites where this mutation is an early occurrence in the progression to carcinoma such as lung (Suzuki *et al.*, 1992; Miller *et al.*, 1992), head and neck (Field *et al.*, 1991, 1994), oesophagus (Hollstein *et al.*, 1991*b*) and bladder (Spruck *et al.*, 1993; Zhang *et al.*, 1994*a*), but not those where p53 is a late occurrence such as stomach (Zhang *et al.*, 1995*a*) and prostate (Zhang *et al.*, 1994*b*). Zhang *et al.* (1995*b*) recently reported no obvious association between tobacco smoking and p53 nuclear expression in colorectal cancer. However, the study had a relatively small sample size and included only patients with Duke's C stage neoplasms.

To investigate the relationship between p53 overexpression and smoking exposure in colorectal cancer, we analysed tumour specimens for p53 protein and categorised them according to the patient's smoking exposure. Case series analysis suggested aetiological heterogeneity for p53 overexpression and smoking exposure and may indicate the presence of distinct causal mechanisms for $p53^+$ cases and $p53^-$ cases. To estimate the individual relative risks of developing a $p53^+$ versus a $p53^-$ tumour we used a healthy control group. We observed that tumours developing through a $p53^+$-dependent pathway were unrelated to smoking exposures. However, those tumours developing through a p53 independent pathway were significantly associated with both smoking status and total pack–years of smoking (trend test: $P = 0.03$). These data may suggest that smoking may initiate tumour mutations early in the adenoma–carcinoma sequence, perhaps in *apc* or *ras* genes (Fearon and Vogelstein, 1990), that do not require p53 mutation/overexpression to progress from an adenoma to a carcinoma. This also may explain conflicting results between risk of colorectal adenomas and carcinoma relative to smoking exposure.

The p53 gene is considered a common target in human carcinogenesis and mutations of this gene may inactivate their encoded mutant proteins. Many studies have observed a high correlation between p53 gene missense mutation and immunohistochemical detection of p53 overexpression (Cordon-Cardo *et al.*, 1994; Umekita *et al.*, 1994).

However, the absence of p53 overexpression does not necessarily imply the absence of p53 mutations, since tumours with frameshift or null mutations may have undetectable levels of p53 protein, although only 8% of all colorectal tumours display this type of alteration (Greenblatt *et al*., 1994). Therefore, misclassification of p53 protein overexpression status as it relates to the underlying mutation is likely to be small in colorectal tumours.

Jones *et al.* (1991) suggested that there are two general patterns of p53 mutations: mutations induced by exogenous carcinogens, and those occurring spontaneously due to endogenous mutagens. Carcinogen-induced mutations are caused by direct interaction of carcinogens with DNA, leading to specific point mutations, predominantly transversions. For example, a study of p53 mutations in non-small cell lung carcinomas found that most mutations were transversions at G and C residues (Chiba *et al*., 1990). This suggests that lung cancer results from the direct interaction of carcinogens in cigarette smoke with DNA.

Spontaneous mutations arise at CpG dinucleotides, which are thought to result from the frequent methylation of the cytosine residue in CpG sites with a high frequency of transitions. These spontaneous mutations are assumed to result from an endogenous process, as no exogenous factors have been identified. In colorectal cancer, most p53 mutations are spontaneous with transitions occurring at CpG sites. However, a small percentage of p53 mutations in colorectal cancer are transversions or occur at non-CpG sites, and may result from an exogenous carcinogen. If we assume that p53 overexpression indicates mutation, it is possible that although the majority of our $p53^+$ cases may be unrelated to smoking (CpG transitions), a small percentage of $p53^+$ tumours (transversions) may be associated with tobacco use. Ideally, future studies should employ immunohistochemical detection of p53 overexpression along with sequence analysis to examine these associations.

The exact mechanism by which cigarette smoking influences colorectal carcinogenesis is unclear. Smoking increases the risk at several cancer sites not directly in contact with smoke including pancreas, stomach, kidney and bladder (US Department of Health, Education, and Welfare, 1979). Carcinogens in smoke could affect the mucosa of the large bowel through direct ingestion or through the circulatory system. Polycyclic aromatic hydrocarbons (PAHs) and heterocyclic amines are two potent carcinogens found in cigarette smoke and are also observed in well-done meat, another risk factor for colorectal cancer (Laden *et al*., 1995).

The possibility exists that cigarette smokers consume a diet consisting of fewer protective and more numerous carcinogenic agents. Smokers generally have lower intakes of fruits and vegetables and higher intakes of fats than non-smokers (Subar and Harlan, 1993). Therefore, the association between p53 (negative) independent tumours and smoking may be confounded by dietary factors associated with this pathway. However in our analysis, a statistically significant dose–response relationship for the association between p53 negative patients and total pack–years of smoking existed (trend test: $P=0.03$), even after adjustment for alcohol, meat, cruciferous vegetables and body mass index. Therefore, it is unlikely that confounding by diet completely accounts for the association between cigarette smoking and the p53 independent pathway.

The use of hospital/screening clinic controls in a case–control comparison may lead to bias since controls may overrepresent healthier lifestyles. However, the odds ratios observed in our case–control comparison are similar to those reported in three recent population-based cohort studies (Giovannucci *et al*., 1994*a* and *b*; Heineman *et al*., 1995). Additionally, the primary use of this control group is to assist in the estimation of the individual relative risks for p53 positive and p53 negative tumour development separately, for smoking exposure variables. This bias is of less concern in our case series analysis since patients are unaware of their p53 status.

The positive consistent finding that smoking increases the risk of colorectal adenoma formation but not cancer has been problematic. Our results suggest that results of studies of colorectal cancer risk and smoking may be affected by inclusion of patients whose tumours harbour p53 genetic alterations that occur late in the adenoma–carcinoma sequence, that are unrelated to smoking history. In addition, smoking was significantly associated in tumours developing through a p53 independent pathway, perhaps the result of initiation of mutations in genes early in the carcinogenic process that do not need a p53 alteration to progress from an adenoma to a carcinoma.

**Acknowledgements**
This research was supported by grants from the National Cancer Institute's Cancer Research Education Training Programs R25 CA18201-19, the Mark Diamond Research Fund and the Nicholas Patterson Perpetual Fund. The authors thank Dwayne Narayan for his assistance with data collection.

### References

BEGG CB AND ZHANG ZF. (1994). Statistical analysis of molecular epidemiology studies employing case-series. *Cancer Epidemiol. Biomarkers Prev*., **3**, 173–175.

BENNETT WP, HOLLSTEIN MC, HE A, ZHU SM, RESAU JH, TRUMP BF, METCALF RA, WELSH JA, MIDGLEY C, LANE DP AND HARRIS CC. (1991). Archival analysis of p53 genetic and protein alterations in Chinese esophageal cancer. *Oncogene*, **6**, 1779–1784.

BRESLOW NE AND DAY NE. (1980). The Analysis of Case Control Studies. In *Statistical Methods in Cancer Research*. Vol. 1. IARC Scientific Publication No. 32. pp. 122–159.

CARTENSEN JM, PERSHAGEN G AND ECKLUND G. (1987). Mortality in relation to cigarette and pipe smoking: 16 years' observation of 25,000 Swedish men. *J. Epidemiol. Commun. Health*, **41**, 166–172.

CHIBA I, TAKAHASHI T, NAU MM, D'AMICO D, CURIEL DT, MITSUDOMI T, BUCHHAGEN DL, CARBONE D, PIANTADASI S, KOGA H, REISSMAN PT, SLAMON DJ, HOLMES EC AND MINNA JD. (1990). Mutations in the p53 gene are frequent in primary, resected non-small cell lung cancer. Lung Cancer Study Group. *Oncogene*, **5**, 1603–1610.

CHOI WY AND KAHYO H. (1991). Effect of cigarette smoking and alcohol consumption in the etiology of cancers of the digestive tract. *Int. J. Cancer*, **49**, 381–386.

CHUTE CG, WILLETT WC, COLDITZ GA, STAMPFER MJ, BARON JA, ROSNER B AND SPEIZER FE. (1991). A prospective study of body mass, height, and smoking on the risk of colorectal cancer in women. *Cancer Causes Control*, **2**, 117–124.

COPE GF, WYATT JI, PINDER IF, LEE PN, HEATLEY RV AND KELLEHER J. (1991). Alcohol consumption in patients with colorectal adenomatous polyps. *Gut*, **32**, 70–72.

CORDON-CARDO C, DALBAGNI G, SAEZ GT, OLIVA MR, ZHANG ZF, ROSAI J, REUTER VE AND PELLICER A. (1994). p53 mutations in human bladder cancer: genotypic versus phenotypic patterns. *Int. J. Cancer*, **56**, 347–353.

DALES LF, FRIEDMAN GD, URY HK, GROSSMAN S AND WILLIAMS SR. (1979). A case control study of relationships of diet and other traits to colorectal cancer in American blacks. *Amer. J. Epidemiol*., **109**, 132–144.

DEMERS RY, NEALE AV, DEMERS P, DEIGHTON K, SCOTT RO AND DUPUIS MH. (1988). Serum cholesterol and colorectal polyps. *J. Clin. Epidemiol*., **41**, 9–13.

DOLL R, GRAY R, HAFNER B AND PETO R. (1980). Mortality in relation to smoking: 22 years' observations on female British doctors. *Br. Med. J*., **280**, 967–971.

DOLL R AND PETO R. (1976). Mortality in relation to smoking: 22 years' observations on male British doctors. *Br. Med. J*., **2**, 1525–1536.

DREXLER J. (1971). Proximal and distal polyp relationships. *Arch. Intern. Med.*, **127**, 466–469.

FEARON ER. (1993). Molecular genetic studies of the adenoma–carcinoma sequence. *Adv. Int. Med.*, **39**, 123–147.

FEARON ER AND VOGELSTEIN B. (1990). A genetic model for colorectal tumorigenesis. *Cell*, **61**, 759–767.

FERRARONI M, NEGRI E, LA VECCHIA C, D'AVANZO B AND FRANCESCHI S. (1989). Socio-economic indicators, tobacco and alcohol in the aetiology of digestive tract neoplasm. *Int. J. Epidemiol.*, **18**, 556–562.

FIELD JK, SPANDIDOS DA, MALLIRI A, GOSNEY JR, YIAGNISIS M AND STELL PM. (1991). Elevated p53 overexpression correlates with a history of heavy smoking in squamous cell carcinoma of the head and neck. *Br. J. Cancer*, **64**, 573–577.

FIELD JK, ZOUMPOURLIS V, SPANDIDOS DA AND JONES AS. (1994). p53 overexpression and mutations in squamous cell carcinoma of the head and neck: expression correlates with the patients' use of tobacco and alcohol. *Cancer Detect. Prev.*, **18**, 197–208.

GARLAND C, BARRETT-CONNOR E, ROSSOF AH, AND PAUL O. (1985). Dietary vitamin D and calcium and risk of colorectal cancer: a 19-year prospective study in men. *Lancet*, **1**, 307–309.

GIOVANNUCCI E, COLDITZ GA, STAMPFER MJ, HUNTER D, ROSNER BA, WILLETT WC AND SPEIZER FE. (1994*a*). A prospective study of cigarette smoking and risk of colorectal adenoma and colorectal cancer in U.S. women. *J. Natl Cancer Inst.*, **86**, 192–199.

GIOVANNUCCI E, RIMM EB, STAMPFER MJ, COLDITZ GA, ASCHERIO A, KEARNEY J AND WILLETT WC. (1994*b*). A prospective study of cigarette smoking and risk of colorectal adenoma and colorectal cancer in U.S. men. *J. Natl Cancer Inst.*, **86**, 183–191.

GRAHAM S, DAYAL H, SWANSON M, MITTELMAN A AND WILKINSON G. (1978). Diet in the epidemiology of cancer of the colon and rectum. *J. Natl Cancer Inst.*, **61**, 709–714.

GREENBLATT MS, BENNETT WP, HOLLSTEIN M AND HARRIS CC. (1994). Mutations in the p53 tumor suppressor gene: clues to cancer etiology and molecular pathogenesis. *Cancer Res.*, **54**, 4855–4878.

HAENSZEL W, BERG JW, SEGI M, KURIHARA M AND LOCKE FB. (1973). Large bowel cancer in Hawaiian Japanese. *J. Natl Cancer Inst.*, **51**, 1765–1779.

HAENSZEL W, LOCKE FB AND SEGI M. (1980). A case–control study of large bowel cancer in Japan. *J. Natl Cancer Inst.*, **64**, 17–22.

HAMMOND EC. (1966). Smoking in relation to the death rates of one million men and women. *NCI Monogr.*, **19**, 127–204.

HAMMOND EC AND HORN D. (1958). Smoking and death rates–report on forty-four months of follow-up of 187,783 men. *JAMA*, **166**, 1294–1308.

HARRIS CC. (1991). Chemical and physical carcinogenesis: advances and perspectives for the 1990s. *Cancer Res.*, **51** (suppl.), 5023s–5044s.

HEINEMAN EF, HOAR ZAHM S, MCLAUGHLIN JK AND VAUGHT JB. (1995). Increased risk of colorectal cancer among smokers: results of a 26-year follow-up of U.S. veterans and a review. *Int. J. Cancer*, **59**, 728–738.

HIGGINSON J. (1966). Etiologic factors in gastrointestinal cancer in man. *J. Natl Cancer Inst.*, **37**, 527–545.

HIRAYAMA T. (1975). Smoking and cancer: a prospective study on cancer epidemiology based on a census population in Japan. Proc. 3rd World Conference on Smoking and Health, New York, June 2–5 1975. Vol. 2. National Cancer Institute: Bethesda, MD.

HOFF G, VATN MH AND LARSEN S. (1987). Relationship between tobacco smoking and colorectal polyps. *Scan. J. Gastroenterol.*, **22**, 13–16.

HOLLSTEIN M, SIDRANSKY D, VOGELSTEIN B AND HARRIS CC. (1991*a*). p53 mutations in human cancers. *Science*, **253**, 49–53.

HOLLSTEIN MC, PERI L, MANDARD AM, WELSH JA, MONTESANO R, METCALF RA, BAK M AND HARRIS CC. (1991*b*). Genetic analysis of human esophageal tumors from two high incidence geographic areas: frequent p53 base substitutions and absence of ras mutations. *Cancer Res.*, **51**, 4102–4106.

HONJO S, KONO S, SHINCHI K, IMANISHI K AND HIROHATA T. (1992). Cigarette smoking, alcohol use and adenomatous polyps of the sigmoid colon. *Jpn. J. Cancer Res.*, **83**, 806–811.

JAIN M, COOK GM, DAVIS FG, GRACE MG, HOWE GR AND MILLER AB. (1980). A case–control study of diet and colorectal cancer. *Int. J. Cancer*, **26**, 757–768.

JAREBINSKI M, ADANJA B AND VLAJINAC H. (1988). Bisocial and other characteristics of the large bowel cancer patients in Belgrade Yugoslavia. *Arch. Geschwulstforsch*, **58**, 411–417.

JAREBINSKI M, ADANJA B AND VLAJINAC H. (1989). Case control study of relationship of some bisocial correlates to rectal cancer patients in Belgrade Yugoslavia. *Neoplasma*, **36**, 369–374.

JONES PA, BUCKLEY JD, HENDERSON BE, ROSS RK AND PIKE MC. (1991). From gene to carcinogen: a rapidly evolving field in molecular epidemiology. *Cancer Res.*, **51**, 3617–3620.,

KABAT GC, HOWSON CP AND WYNDER EL (1986). Beer consumption and rectal cancer. *Int. J. Epidemiol.*, **15**, 494–501.

KAHN HA. (1966). The Dorn study of smoking and mortality among U.S. veterans: report of eight and one-half years of observation. *NCI Monogr.*, **19**, 1–126.

KIKENDALL JW, BOWEN PE, BURGESS MB, MAGNETTI C, WOODARD J AND LANGENBERG P. (1991). Cigarettes and alcohol as independent risk factors for colonic adenomas. *Gastroenterology*, **26**, 758–762.

KLATSKY AL, ARMSTRONG MA, FRIEDMAN GD AND HIATT RA. (1988). The relations of alcoholic beverage use to colon and rectal cancer. *Amer. J. Epidemiol.*, **128**, 1007–1015.

KONO S, IKEDA M, TOKUDOME S, NISHIZUMI M AND KURATSUNE M. (1987). Cigarette smoking, alcohol, and cancer mortality: a cohort study of male Japanese physicians. *Jpn. J. Cancer Res. (Gann)*, **78**, 1323–1328.

KUNE GA, KUNE S, VITETTA L AND WATSON LF. (1992*a*). Smoking and colorectal cancer risk: data from the Melbourne Colorectal Cancer Study and brief review of literature. *Int. J. Cancer*, **50**, 369–372.

KUNE GA, KUNE S, WATSON LF AND PENFOLD C. (1992*b*). Smoking and adenomatous polyps (letter). *Gastroenterology*, **103**, 1370–1371.

LAYTON DW, BOGEN KT, KNIZE MG, HATCH FT, JOHNSON VM AND FELTON JS. (1995). Cancer risk of heterocyclic amines in cooked foods: an analysis and implications for research. *Carcinogenesis*, **16**, 39–52.

LANE DP. (1990). Mutation of the p53 gene and accumulation of the p53 protein: common steps found in the majority of human cancers. *Accomplishments in Cancer Res.*, 252–266.

LEE WC, NEUGUT AI, GARBOWSKI GC, FORDE KA, TREAT MR, WAYE JD AND FENOGLIO-PREISER C. (1993). Cigarettes, alcohol, coffee and caffeine as risk factors for colorectal adenomatous polyps. *Ann. Epidemiol.*, **3**, 239–244.

MARTINEZ ME, MCPHERSON RS, ANNEGERS JF AND LEVIN B. (1995). Cigarette smoking and alcohol consumption as risk factors for colorectal adenomatous polyps. *J. Natl Cancer Inst.*, **87**, 274–279.

MARTINEZ I, TORRES R, FRIAS Z, COLON JR AND FERNANDEZ N. (1981). Factors associated with adenocarcinomas of the large bowel in Puerto Rico. *Rev. Latinoam Oncology Clin.*, **13**, 13–20.

MILLER CW, SIMON K, ASLO A, KOK K, YOKOTA J, BUYS CH, TERADA M AND KOEFFLER HP. (1992). p53 mutations in human lung tumors. *Cancer Res.*, **52**, 1695–1698.

MONNET E, ALLEMAND H, FARINA H AND CARAYON P. (1991). Cigarette smoking and the risk of colorectal adenoma in men. *Scand. J. Gastroenterol.*, **26**, 758–762.

OLSEN J AND KRONBERG O. (1993). Coffee, tobacco, and alcohol as risk factors for cancer and adenoma of the large intestine. *Int. J. Epidemiol.*, **22**, 398–402.

PAPDIMITRIOU C, DAY N, TZONOU A, GEROVASSILIS F, MONOUSOS O AND TRICHOPOULOS D. (1984). Bisocial correlates of colorectal cancer in Greece. *Int. J. Epidemiol.*, **13**, 155–159.

PETERS RK, GARABRANT DH, YU MC AND MACK TM. (1989). A case control study of occupational and dietary factors in colorectal cancer in young men by subsite. *Cancer Res.*, **49**, 5459–5468.

ROGOT E AND MURRAY JL. (1980). Smoking and causes of death among U.S. veterans: 16 years of observation. *Publ. Health Rep.*, **95**, 213–222.

SANDLER RS, SANDLER DP, COMSTOCK GW, HELSING JT AND SHORE DL. (1988). Cigarette smoking and the risk of colorectal cancer in women. *J. Natl Cancer Inst.*, **80**, 1329–1333.

SANDLER RS, LYLES CM, MCAULIFFE C, WOOSLEY JT AND KUPPER LL. (1993). Cigarette smoking, alcohol and the risk of colorectal adenomas. *Gastroenterology*, **104**, 1445–1451.

SHI SR, KEY MC AND KALRA KL. (1991). Antigen retrieval in formalin-fixed, paraffin-embedded tissues: an enhancement method for immunohistochemical staining based on microwave oven heating of tissue sections. *J. of Histochem. Cytochem.*, **39**, 741–748.

SLATTERY ML, WEST DW, ROBISON LM, FRENCH TK, FORD MD, SCHUMAN KL AND SORENSON AW. (1990). Tobacco, alcohol, coffee, and caffeine as risk factors for colon cancer in a low risk population. *Epidemiology*, **1**, 247–253.

SPRUCK CH, RIDEOUT WM, OLUMI AF, OHNESEIT PF, YANG AS, TSAI YC, NICHOLS PW, HORN T, HERMANN GG, STEVEN K, ROSS RK, YU MC AND JONES PA. (1993). Distinct pattern of p53 mutations in bladder cancer: relationship to tobacco usage. *Cancer Res.*, **53**, 1162–1166.

STAZEWSKI J. (1969). Smoking and cancer of the alimentary tract in Poland. *Br. J. Cancer*, **23**, 247–253.

SUBAR AF AND HARLAN LC. (1993). Nutrient and food group intake by tobacco use status: the 1987 National Health Interview Survey. *Ann. NY Acad. Sci.*, **686**, 310–321.

SUZUKI H, TAKAHASHI T, KUROISHI T, SUYAMA M, ARIYOSHI Y, TAKAHASHI T AND UEDA R. (1992). p53 mutations in non-small cell lung cancer in Japan: association between mutations and smoking. *Cancer Res.*, **52**, 734–736.

TAJIMA K AND TOMINAGA S. (1985). Dietary habits and gastrointestinal cancers: a comparative case control study of stomach and large intestinal cancers in Nagoya, Japan. *Jpn. J. Cancer Res.*, **76**, 705–716.

TUYNS AJ, PEQUIGNOT G, GIGNOUX M AND VALLA A. (1982). Cancers of the digestive tract, alcohol and tobacco. *Int. J. Cancer*, **30**, 9–11.

TVERDAL A, THELLE D, STENSVOLD I, LEREN P AND BJARTVEIT K. (1993). Mortality in relation to smoking history: 13 years' follow-up of 68,000 Norwegian men and women 35–49 years. *J. Clin. Epidemiol.*, **46**, 475–487.

UMEKITA Y, KOBAYASHI K, SAHEKI T AND YOSHIDA H. (1994). Nuclear accumulation of p53 protein correlates with mutations in the p53 gene on archival paraffin-embedded tissues of human breast cancer. *Jpn. J. Cancer Res.*, **85**, 825–830.

US DEPARTMENT OF HEALTH, EDUCATION, AND WELFARE. (1979). *Smoking and Health. A Report to the Surgeon General.* DHEW Publication no. PHS 79-50066. US Govt Print Office: Washington DC.

VOBECKY J, CARO J AND DEVROEDE G. (1983). A case control study of risk factors for large bowel carcinoma. *Cancer*, **51**, 1958–1963.

WEIR JM AND DUNN JE JR. (1970). Smoking and mortality: a prospective study. *Cancer*, **26**, 105–112.

WILLIAMS RR, SORLIE PD, FEINLEIB M, MCNAMARA PM, KANNEL WB AND DAUBER TR. (1981). Cancer incidence by levels of cholesterol. *J. Amer. Med. Assoc.*, **245**, 247–252.

WILLIAMS RR AND HORM JW. (1977). Association of cancer sites with tobacco and alcohol consumption and socioeconomic status of patients: interview study from the Third National Cancer Survey. *J. Natl Cancer Inst.*, **58**, 525–547.

WU AH, PAGANINI-HILL A, ROSS RK AND HENDERSON BE. (1987). Alcohol, physical activity and other risk factors for colorectal cancer: a prospective study. *Br. J. Cancer*, **55**, 687–694.

WYNDER EL AND SHIGEMATSU T. (1967). Environmental factors of cancer of the colon and rectum. *Cancer*, **20**, 1520–1561.

WYNDER EL, KAJITANI T, ISHIKAWA S, DODO H AND TOKANO A. (1969). Environmental factors of cancer of the colon and rectum. II: Japanese epidemiological data. *Cancer*, **23**, 1210–1220.

ZAHM SH, COCCO P AND BLAIR A. (1991). Tobacco smoking as risk factor for colon polyps. *Amer. J. Public Health*, **81**, 846–849.

ZHANG ZF, SARKIS AS, CARDON-CARDO C, DALBAGNI G, MELAMED J, APRIKIAN A, POLLACK D, SHEINFELD J, HERR HW, FAIR WR, REUTER VE AND BEGG C. (1994*a*). Tobacco smoking, occupation and p53 nuclear overexpression in early stage bladder cancer. *Cancer Epidemiol. Biomarkers Prev.*, **3**, 19–24.

ZHANG ZF, APRIKIAN A, SARKIS AS, ZENG Z, POLLACK D, CARDON-CARDO C, FAIR WR AND BEGG C. (1994*b*). Factors associated with p53 nuclear accumulation in prostate adenocarcinoma. *Int. J. Oncol.*, **4**, 897–902.

ZHANG ZF, KARPEH MS, LAUWERS GY, MARRERO AM, POLLACK D, CARDON-CARDO C AND BEGG C. (1995*a*). A case-series study of p53 nuclear overexpression in early stage intestinal-type stomach cancer. *Cancer Det. Prev.*, **2**, 156–164.

ZHANG ZF, ZENG ZS, SARKIS AS, KLIMSTRA DS, CHARYTONOWICZ E, POLLACK D, VENA J, GUILLEM J, MARSHALL JR, CORDON-CARDO C, COHEN AM AND BEGG CB. (1995*b*). Family history of cancer, body weight and p53 nuclear over-expression in Duke's C colorectal cancer. *Br. J. Cancer*, **71**, 888–893.